# Recent Advances in Detecting Mitochondrial DNA Heteroplasmic Variations

**DOI:** 10.3390/molecules23020323

**Published:** 2018-02-03

**Authors:** Mengqin Duan, Jing Tu, Zuhong Lu

**Affiliations:** State Key Lab of Bioelectronics, School of Biological Science and Medical Engineering, Southeast University, Nanjing 210096, China; 220163884@seu.edu.cn

**Keywords:** heteroplasmy, mtDNA, mitochondria, next generation sequencing

## Abstract

The co-existence of wild-type and mutated mitochondrial DNA (mtDNA) molecules termed heteroplasmy becomes a research hot point of mitochondria. In this review, we listed several methods of mtDNA heteroplasmy research, including the enrichment of mtDNA and the way of calling heteroplasmic variations. At the present, while calling the novel ultra-low level heteroplasmy, high-throughput sequencing method is dominant while the detection limit of recorded mutations is accurate to 0.01% using the other quantitative approaches. In the future, the studies of mtDNA heteroplasmy may pay more attention to the single-cell level and focus on the linkage of mutations.

## 1. Introduction

Mitochondria are vital organelles of most eukaryotic cells, which play an important role in cellular energy supply, called ‘powerhouses of the cell’. Mitochondrial DNAs (mtDNA) form double-strand circular molecules which contain about 16,569 DNA base pairs, including a D-loop region, 13 protein encoding genes, two rRNA (12S rRNA and 16S rRNAs) and 22 tRNA encoding regions [1]. Because of the maternal inheritance feature of mitochondrion, mtDNA sequencing has become a generally method of human evolutionary line studies. A plenty of diseases like Leber’s hereditary optic neuropathy (LHON), mitochondrial encephalopathy, lactic acidosis and stroke-like episodes (MELAS), myoclonic epilepsy and ragged-red fibres (MERRF), chronic progressive external ophthalmoplegia (CPEO) and breast cancer (mostly affected by mutations in d-loop region [2]) have been detected to be associated with mtDNA’s mutation [3], revealing that the disfunction of mtDNA are relevant. Therefore, mitochondrial genome is a significant object of researches in clinical diagnosis.

Generally speaking, one mitochondrion can harbor 2–10 mtDNA molecules [4], and the number of mitochondria in a cell differs from various tissues or organs according to the energy demands of them. Additionally, without the protection of histone, mtDNA is highly mutable, with about 6–17-fold higher mutation rate than nuclear DNA [5]. In consequence of that, the normal mtDNA molecules and the mutated ones are often mixed up and display a ‘co-existence’ situation, termed heteroplasmy [6]. 

**Figure 1:**
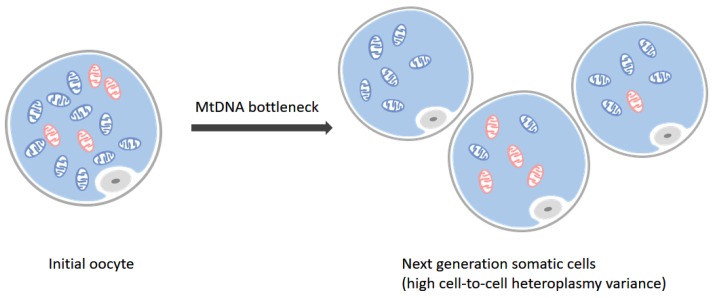
Bottleneck effect. There are two types of mtDNA in initial oocyte (**left**), where blue stand for mitochondria harboring wild-type mtDNA and red ones represent mutated ones. During the meiosis acting under the mtDNA bottleneck effect, the mitochondria in next generation somatic cells take on a high cell-to-cell heteroplasmy variance situation (**right**). Some of them may harbor all wild-type mtDNA while others accumulate one or more mutations.

As far back as 1985, researchers had detected that more than one mitochondrial genome haplotype was present in an individual [7,8,9,10,11]. Most of these early studies focused on the inter-individual divergences between members of a pedigree, thus the uneven sampling of mtDNA from one generation to their progenies was found and discussed as a term ‘bottleneck’ (Figure 1). This is one of widely appreciated sources of mtDNA heteroplasmy by means of heredity. Another heteroplasmy headstream is considered to be transmission of paternal mtDNA during fertilization.

A well-known case was published in 2002 that a man with mitochondrial myopathy harbored 90% of mitochondrial genome in skeletal muscles which was the same as his father’s [12]. Actually, there existed co-localization between some hotspots of mitochondrial genome breakpoints and 7S DNA region in this case. Hence it was possible that the recombination of parental mtDNA originated from ‘template switching’ of 7S DNA [13]. However, compared to the rare paternal-origin situation, de novo mutations drew more attention. On account of the lesser proteins’ protection than nuclear genome, mtDNA faces higher risk of damages which can lead to errors during DNA replication. The free radical theory, which attributes the accumulation of heteroplasmic variations to mtDNA damages during the aging process, is widely accepted. This theory considers mitochondria to be the major sources of cellular reactive oxygen and nitrogen species (RONS) which result in further damage to mtDNA [14]. Electrons leaking from electron transfer chain (ETC) activity arisen in mitochondrial matrix are the beginning of mtDNA damage’s ‘vicious cycle’ [15]. Then the amount of reactive oxygen species (ROS) production like O^2−^ arose by electrons. After that, as the absence of effective mtDNA transcription products caused by ROS production, the descending of ETC activity leads to a higher possibility of electrons leaking [15]. 

In accordance with studies of mitochondrial damages, point mutations do accumulate with aging in human mtDNA [16,17,18]. In response to the lesions throughout aging, mitochondria can repair themselves. They transform from reticular nets form as ‘chain state’ into individual fashion through fusion and fission [14]. Then several means of mitophagic activity like selective removal of signaling damaged mitochondria help maintain the proportion of wild-type mtDNA molecules to keep functional stability [19]. 

In pace with developing of mtDNA heteroplasmy studies, intra-individual heteroplasmic variations were detected [5] and the discovered large span of heteroplasmic level between different tissues verified the bottleneck phenomenon during heredity of mtDNA [20]. The analysis of heteroplasmy levels from different germinal layers might explain that the formation and spread of mutations occurred discrepantly during embryonic development stages after amphigenesis. And the results indicated that the updating ability of the tissues like hemocytes or bone marrow may eliminate those mutated mtDNA molecules. In contrast, these ‘post-mitotic’ tissues with a slower rate of metabolism keep the mutations which may turn to be pathogenic sources. Hence, even though the genotypes of mtDNA extracted from blood showed high homogeneity, the significant heteroplasmy was still detected in hairs without roots from a single individual [21]. Li et al.’s studies involved more specimens [18]. They obtained more tissue-specific results, then discussed the selective pressure of those heteroplasmic mutations among different tissues. It is found that the development of mtDNA mutations’ tissue-specific pattern is often concerned with the tissue-specific metabolic rate, cycling of cells and the demand of bioenergy. As a consequence of that, tissues or organs possessing higher rate of mitochondrial turnover are prone to process positive selection to retain the state of ‘survival of slowest’ while others tend to random genetic drift [18,22]. 

Concurrent with the researches of heteroplasmy of mtDNA between tissues, intracellular mutations offered more detailed information. Most of these single-cell studies paid attention to the diversities of several particular variations like disease-causing genes and amplified these fragments harboring mutations before sequencing them. Some of these investigations instituted comparisons of mtDNA heteroplasmic rate between single cell and bulk cells in tissues [23]. They confirmed that clonal expanded mtDNA mutations which started from single mutated molecules were as well as abundant in individual cells [16,17]. Consistent with the tissue-specific study results, segregation of mtDNA heteroplasmic units contributed to the higher heteroplasmy rate of post-mitotic cells [24,25]. Nevertheless, explorations of organelles still have great room for development. Caverlier et al. obtained 114 mitochondria from fibroblasts through fluorescence-activated cell sorting (FACS) device. And they learned that approximately a quarter of mitochondria harbored a mixture of wild-type and mutated mtDNA. Close to the proportion 0.58 in bulk cells, mutated mtDNA molecules accounted for 0.48, showing typically process of homogenization among mitochondria [22]. 

To go deep into studies of the mitochondrial genome, more detailed and accurate mtDNA sequences should be collected. Along with the development of sequencing technologies, those requirements are responded commendably. However, due to the polymorphism of mtDNA, heteroplasmy calling especially for low-level heteroplasmic variance is still a challenge. Due to the small scale of mitochondrial genome, some larger exogenous genomes especially nuclear genome might disturb distinguishing mtDNA heteroplasmy exactly. Thus, enriching mitochondrial genome have been regarded as an essential step before heteroplasmy calling. In this review, we list several approaches to mtDNA’s heteroplasmy research, including enrichment methods and heteroplasmy calling methods.

## 2. Methods of Enriching mtDNA

**Figure 2:**
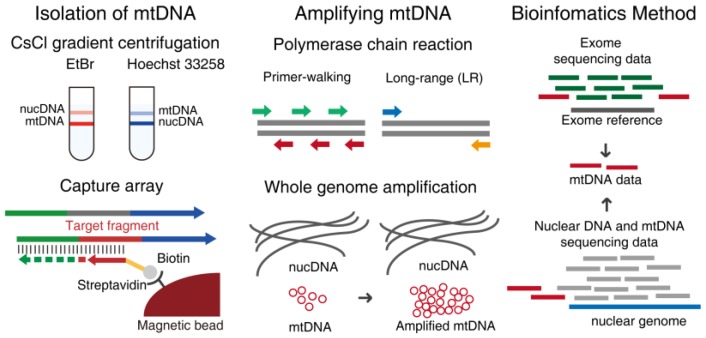
A brief schematic diagram of enriching methods for mtDNA. Isolation methods separate or capture mtDNA from specimen directly, which need abounding biospecimen. Amplifying mtDNA methods whose mainly approaches are PCR and WGA, make the requirement for amount and freshness of biospecimen more broaden, and mtDNA can be amplified specifically. Bioinformatics methods can dig mtDNA data from other relevant genome sequencing data like exome and whole genome in cells.

According to whether amplification means were used or not, we divided the various enrichment methods into two main categories, isolation and amplification. However, isolating mtDNA from biospecimens risks contamination from the nuclear genome, especially paralogous fragments of mtDNA. In consideration of the high copy number of mtDNA molecules compared with nuclear genome, contamination would not have a great effect on the detection of heteroplasmic variations with higher heteroplasmy frequency and the impact of nuclear DNA should be taken into account when identifying low level heteroplasmies as accurately as possible. Thus, we summarized several bioinformatics methods in a separate section which discussed some strategies to minimize the influence of contamination (Figure 2). 

### 2.1. Isolation of mtDNA

Methods of isolation focus on the separation of organelles, and then obtain intact mitochondria in an early stage. Now the mitochondria extracted from biospecimens are required to maintain intact form and biological activity even the function of respiration action according to their research purposes [26,27]. In this review, we concerned more about the yield and quality of mtDNA, and less involved the extraction of mitochondria. 

#### 2.1.1. Density Gradient Centrifugation

In earlier times, investigators utilized differential centrifugation to separate certain organelles from cells to extract DNA from them. As early as 1968, Bielka et al. separated mitochondria from eggs by CsCl density centrifugation [28]. This method makes use of a neutral medium to establish a density gradient environment, where the organelles float or sink depending upon their densities when centrifuged. CsCl was widely employed as a typical neutral medium, which could prevent water gain or loss by the organelles and keep mitochondria in good shape. As for the cell disintegration step before centrifugation, digitonin is an ideal reagent for this purpose. Owing to the ability of combing with free cholesterol, digitonin could disrupt cellular membranes readily. Mitochondria isolated by digitonin showed good preservation of respiratory function, because there was little or no cholesterol in the mitochondrial inner membranes. With the aid of this reagent, the amount of biosample could be reduced to about 25 mg [29]. However, digitonin had been found to modify the responses of brain mitochondria contributing to the induction of apoptosis, so the use of digitonin should be compatible with downstream studies. 

Obviously, density gradient centrifugation greatly decreases the quality of nuclear DNA and avoids the interference of other mitochondrial homologous genes. However, this method was costly and time-consuming, and also required a large quantity of tissue samples, which limited the its widespread use. Except for extraction of mitochondria firstly, mtDNAs could directly be isolated depending on density differences. There were two kinds of dyes, ethidium bromide (EtBr) and Hoechst 33,258, which could insert between the purine and pyrimidine base pairs or bound to the large groove of mtDNA molecules. They would choose different genomes to bind according to the AT:GC ratio. The insertion of dyes also let the supercoiled DNA binds relaxed, which might cause the decrease of DNA’s density, helping separate mtDNA from nuclear DNA easier [30]. However, taking the hardship of obtaining mitochondria as well as small-scale of mitochondrial genome into consideration, researchers enriched the mtDNA from specimens through amplifying methods instead. 

#### 2.1.2. Capture Array 

Capture of mtDNA fragments from cracked bio-samples directly is also a frequent approach. Either used in solution or on a surface, the probes for capturing targets must be purchased from their manufacturers, which might cost a lot. Researchers would use other fragments to replace the custom probes. Briggs et al. developed a method termed primer extension capture (PEC) method where high specificity primers and a DNA polymerase was used to capture target sequences from an adaptor-ligated DNA library [31]. Maricic et al. presented a way where long range PCR products were made single-stranded as capture probes to hybridize with pooled sequencing library fragments. As a solid support, streptavidin-coated magnetic beads would capture biotinylated baits. The sample in their experience was a mixture of 46 individuals, and all the 46 mitochondrial whole genomes were captured successfully with depth was at least 80× [32]. In this way the cost of designing probes and chip was saved. Noticing the advantage of distinguishing multi-samples in parallel, capture enrichment method was utilized by Liu et al. for mitogenomics biodiversity analysis of insect. Probes they designed was based on mitochondrial protein-coding scaffolds from 1000 Insect Transcriptome Evolution project (1KITE) [33]. After a series of probe-selecting calculations with the consideration of melting temperature and the mismatch tolerance, they selected 73-mers as the proper length of probes. Eventually, the proportion of mtDNA was increased by approximately 100-fold, from 0.47% to 42.52% [33]. Nowadays, capture methods are more applied in the area of ancient germ line studies like investigating the population history of some groups of humans [34], and mtDNA have an advantage over nuclear DNA while sequencing the nucleotide samples extracted from fossils or bones, which are often highly degraded [35]. 

### 2.2. Amplifying mtDNA 

Directly capture methods often call for enough amount and freshness of the biospecimen, and display disadvantages of efficiency. As a result, amplifying mtDNA molecules can increase the content of mtDNA and cut down laborious experiments. 

#### 2.2.1. Polymerase Chain Reaction

Polymerase Chain Reaction (PCR) is one of frequently-used approaches to amplify DNA specimens, especially for enrichment of small-scale genome like mtDNA. There is a widely utilized PCR method called ‘primer-walking’ which requires two or more pairs of PCR primers with overlapping regions between each of these amplicons [36]. The primer-walking method was constantly used to splice amplicons together into entire mitochondrial genome sequence. Although PCR could amplify the targeted regions purposively, while coming to the process of heteroplasmy calling in primer binding regions, this method brought confusions, as the degree of coverage between the overlapped and un-overlapped region in products was vary widely. Zhang et al. developed a long-range PCR (LR-PCR) method which could amplify the whole genome of mtDNA with one pair of primers in one step which resolved this problem to some extent. To demonstrate the uniformity of the LR-PCR method, they also designed a normal PCR with 24 pairs of primers to amplify 14 different nuclear gene loci [37]. Then the products delivered from two approaches were respectively sequenced by massive parallel sequencing (MPS). As a consequence, the sequencing results revealed that the coverage of those multi-primers products is uniform within each PCR fragment itself, but much less than the overlapping regions while the LR-PCR giving a well-proportioned coverage throughout the entire mitochondrial genome proved by deep sequencing. 

However, coexisting with the convenience brought by PCR, biases cannot be ignored. For instance, if the primer-binding region harbors base alteration, PCR primers would readily coalesce homologous regions of mtDNA in the nuclear genome rather than the correct binding sites [38]. Hence it is imperative to employ specific primers which exclusively combine to mtDNA as well as polymerase with high fidelity. PCR amplification bias would also disturb during library preparation. Aird et al. found that the PCR temperature ramping speed will affect loci with extreme base compositions such as GC-rich regions [39]. They researched base-composition bias by following the Illumina (Illumina Inc., San Diego, CA, USA) standard library construction protocol, focusing on an artificial GC-rich genome. After sequencing the PCR-free against PCR-amplified libraries and optimizing the PCR conditions, it was obvious that the faster the PCR temperature ramping speed, the less abundance of high-GC-content amplicons. 

#### 2.2.2. Whole Genome Amplification 

Whole genome amplification (WGA), a method for robust amplification of an entire genome, including PCR-based and isothermal-based amplification method, is a powerful tool of amplifying DNA molecules from minute quantities to amounts that can be used for sequencing studies. 

PCR-based WGA includes primer-extension pre-amplification (PEP) [40], degenerate oligonucleotide primed PCR (DOP-PCR) and ligation-mediated PCR (LM-PCR), while the usual isothermal-based approaches, containing multiple displacement amplification (MDA) as well as multiple annealing and looping-based amplification cycles (MALBAC) [41]. For MDA, DNA amplification is carried out by Φ29 DNA polymerase, a high fidelity enzyme, which is able to reduce the error rate greatly. Compared to PCR-based methods, MDA have no DNA template tendentiousness and the 3′-5′ proofreading activity of Φ29 polymerase greatly guarantees the sensitivity during amplification. Thus, WGA technologies are able to expand the tiny amount of nucleic acid samples accurately such as degraded DNA which means this method is suitable for forensic casework [42,43]. The first application to the research of whole mitochondrial genome amplifying was a commercial REPLI-g midi kit (Qiagen Inc., Duesseldorf, Germany) [44]. This MDA-based kit was not originally intended to amplify mtDNA, but the analysis of products showed that mtDNA was amplified about 133-fold. The cancer associated mutations between the native mtDNA and WGA amplified material were consistent with a level of 99.995%. Other than an unbiased amplification of whole genome, there were 13 new heteroplasmies identified in WGA amplified DNA. After that, mitochondrial specific amplification kit REPLI-g Mitochondrial DNA Kit (Qiagen Inc.) was utilized to amplify mitochondrial genome exclusively and to separate mtDNA from linear DNA molecules. Serial dilution before amplification would help increase the specificity of mitochondrial genome [45]. Owing to high efficiency of mtDNA amplification, researches of haplogroup were also employing this technology. And investigators found a correlation between sub-haplogroup H5 and high risk of Alzheimer’s disease (AD) [46]. Nevertheless, in the by-products of MDA it has been noticed that some chimera fragments might be produced due to the strand displacement of Φ29 polymerase [47], but in view of the small scale of the mitochondrial genome, we don’t think chimera reads will have a great influence on mtDNA analysis. 

#### 2.2.3. Bioinformatics Methods 

The methods mentioned above are all about enriching mitochondria or mtDNA directly. However, due to the feature that the majority of mitochondrial genome is gene-coding regions, some indirect approaches such as digging mtDNA sequences from exome sequencing data are also attainable. Exome data comes from all exon capture and sequencing, that is there is a huge possibility that mtDNA fragments are obtained at the same time. A research revealed that there existed some unintentionally sequenced reads including non-coding regions like introns or UTRs and mitochondrial genome discovered from exome data [48]. As previously known, the sequences of nuclear DNA and mtDNA are not independent of each other. There might be as long as ~1 M paralogous fragments of mtDNA called nuMTs (nuclear-mitochondrial) in nuclear genome [49]. NuMTs offer a snapshot of the evolutionary process from the incorporation of an α-proteobacterium into a larger eukaryotic cell 1.8 billion years ago [45]. In consequence, while designing probes or baits for capturing exon fragments, some mtDNA must be acquired which researchers used to regard as contamination [50]. Nowadays, a number of studies have verified that it is feasible to mine mitochondrial genomes from exome sequencing data [48,51]. According to a feasibility study of Zhang et al., these off-target data turn out to be a reliable source of mitochondrial genome reads with an about 100× depth of mitochondrial genome compared to the data from RNA sequencing, showing a more uniform coverage result [52]. 

Despite the lower intensity of lab work this method brings, paralogous fragments turned out to be the barrier to the precision of heteroplasmy calling. Approximately 0.03% of nuclear genome is nuMTs, which can be mapped to mitochondrial reference sequence [38]. These nuMTs should be removed carefully for fear that heteroplasmic fraction (HF) determination will be interfered by them. Some cases disturbed by nuMTs can be identified as ‘piggyback’ peaks in sequencing spectra which is two different base signals with inconsistent peak heights presenting at a single loci [38,49]. To get over this hurdle, three mapping strategies of mtDNA from exon sequencing data have been compared. The most efficient one is mapping the data against the nuclear reference and mitochondrial reference simultaneously, then abandoning those multi-mapped data [48]. However, due to the request of more accurate low-level heteroplasmy detection, extracting mtDNA from exome data may not satisfy the demand of sequencing depth commendably. Considering the cost of next generation sequencing decreasing, researchers may prefer accessing next generation sequencing (NGS) technology nowadays.

## 3. Methods of Detecting Heteroplasmy

Considering the detection limit and variations type of heteroplasmy detecting approaches, we list a table (Table 1) to briefly discuss the topic. 

### 3.1. Sanger Sequencing

**Figure 3:**
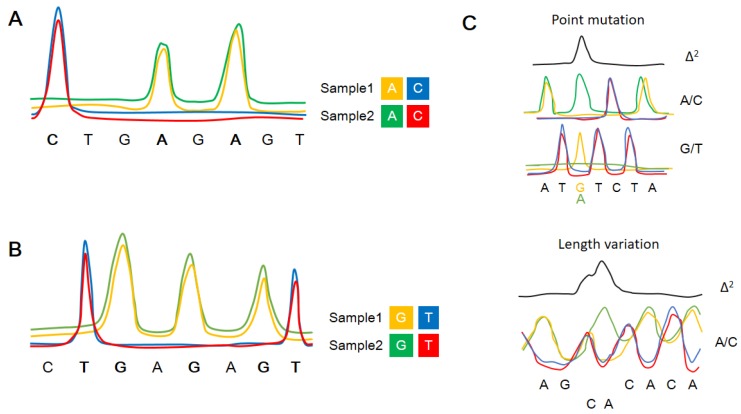
A schematic diagram of polymorphism ratio sequencing (PRS). (**A**,**B**) present the four-color labeling scheme for A/C and G/T in different samples. (**C**) shows the Δ^2^ plot generated by squaring the differences between two samples, including the detection of point mutations and short length variations . The resolution of Δ^2^ plot (peak height) is about 5%.

In 1977, Frederick Sanger and his colleagues developed a DNA sequencing method which based on chain-terminating dideoxynucleotides during DNA replication in vitro. This method has been the most widely used one for approximately 25 years. Nowadays, Sanger sequencing is still a frequently-used approach while studying smaller-scale project because of its speed. However, the poor quality of the first 15~40 bp sequence with the Sanger method is a common challenge due to the influence of the PCR primers as mentioned above. Naue et al. sequenced 883 samples from nine different tissues of 100 unrelated individuals by Sanger sequencing, mini-sequencing and massive parallel sequencing, then the results showed an unexpectedly high number of heteroplasmies using Sanger sequencing [5], but as for the discovery of low-level heteroplasmy, Sanger sequencing might be slightly inferior. As Zhang et al. put forward, the lowest heteroplasmy ratio detected by the Sanger sequencing was around 15% [37]. A new approach named polymorphism ratio sequencing (PRS) was developed by Blazej et al. for single nucleotide polymorphism discovery and genotyping based on some modifications of traditional Sanger sequencing (Figure 3). They applied one kind of nucleotide every sequencing run, then they squared the sum of the differences between the sample and reference traces for all four comparisons. They collected the data of whole mitochondrial genome with the aid of microfluidic device called micro capillary array electrophoresis (μCAE) [54] and drew a circle plot of all the polymorphisms analyzed with the lowest heteroplasmic level of ~5% [55]. Nevertheless, this method, which based on the analysis of spectrum, was readily disturbed by systematic errors. It offered a rough conclusion of qualitative analysis rather than an accurate quantitative measurement for heteroplasmic variations.

Sanger sequencing nowadays is still a gold standard of hateroplasmic variation detection despite its comparatively lower heteroplasmy detection limit [56]. As a consequence, while referring to the point mutation or detecting the rarely low ratio of heteroplasmy, investigators prefer methods with higher sensitivity and specificity [37]. 

### 3.2. Microarray-Based Methods

Some studies showed that heteroplasmy occurred tending to be concentrated in certain regions called mutational hotspots [5]. Hence methods based on high-throughput capture arrays will do well in studying recorded mutational hotspots. Custom-designed single strand DNAs (ssDNAs) called oligonucleotide probes are attached to a solid surface forming a microarray chip, and then those probes hybridize to the fragments to be detected. Sigurdsson et al. utilized a multiplex tag-array minisequencing method to detect 150 single nucleotide polymorphisms (SNPs) in two hypervariable regions (HVR) I and II of the mitochondrial genome [57]. As a consequence, they got a significant high overall success rate of genotyping (95.3%). The failures came from poor targeted DNA quality, primer-biding sites and random failures during the process of hybridization. 

Affymetrix designed a sequencing microarray named MitoChip, which was used for the detecting heteroplasmic variation position of mitochondrial genomes as a tool for early detection of cancer [58]. This tool allowed for sequencing >29 kb of double-stranded DNA in a single assay. Then the MitoChip v2.0 was developed with the GSEQ 4.1 algorithm that participated heteroplasmy calling modified. Thus the detectable rate of MitoChip was raised to 99.75%, as well the rate of accuracy was reach up to 99.988% [59].

Compared to Sanger sequencing, the microarray methods have a significant feature of high-throughput with the advantage of shorter test period and less cost, which can be applied in the field of clinical diagnosis. Capture arrays showed their convenience and efficiency in single nucleotide determination, but are unable to provide information about undiscovered abnormal loci because the design of oligonucleotide probes has to following the existing sequences. Custom capture arrays can just obtain judgments about the existence of mutations, without exactly knowing the quantified ratio of the heteroplasmic locus [60].

### 3.3. PCR-Based Methods

Except for common amplification functions, PCR also allows some subtle changes to meet the quantitative demands with the participation of some kinds of fluorochrome or endonuclease. Quantitative real-time PCR (qPCR) is able to monitor the amount of DNA molecule being amplified in real time, so it helps determine the copy number of mtDNA in a single cell [61], and other mtDNA disfunctions like large-scale deletion [62].

**Figure 4:**
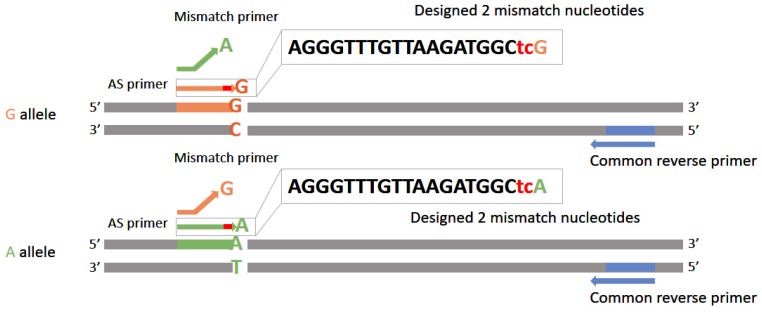
The design of primers in ARMS-qPCR. A set of primers in ARMS is composed of 3 primers, including one common revers primer (blue) and two allele-specific (AS) forward primers (orange and green). The last base of AS primers are set purposively to be consistent with altered bases, while the two nucleotides immediately 5’ to the mutated site (marked as red) are also changed to increase the resolving power of ARMS.

Amplification Refractory Mutation System Quantitative PCR (ARMS-qPCR) is a kind of real-time PCR technology with two upstream primers and one downstream primer. Modification of one or more bases’ upstream primer makes this method possess a more sensitive heteroplasmy detection ability [63]. By quantitatively analyzing the proportion of mutated and wild-type DNA genotypes, the last base of the forward primer would be changed into the corresponding nucleotide which was able to form complementary base-pairs of mutated and wild-type DNA molecules, respectively (Figure 4). However, the binding capacity between PCR primers and templates as well as the amplification efficiency might not be affected much by changing the last base so that the actual proportion of single nucleotide polymorphisms (SNPs) could not be distinguished precisely. Hence several mismatches would be introduced into primers to enhance the specificity of recognizing the wild-type and the mutated bases. It had been shown that introducing two nucleotides immediately 5’ to the mutation site made the detection limit of recorded variations rise to 0.1% [63]. 

During the same time when PCR technologies are wildly utilized, as mentioned before, PCR artifacts are indistinguishable from genuine mtDNA mutations. Single-molecule PCR (smPCR), which separates the mutated and wild-type DNA molecules before amplified, has burgeoned for this reason. This approach conducting a molecule-by-molecule mode avoids too much effort being spent on nonproductive sequencing of the wild-type DNA. Due to the low abundance of template DNA, smPCR products can exclude the interferences resulting from jumping PCR or allelic preference. PCR errors can be easily identified upon direct sequencing of products as low-intensity subpeaks in the spectrum [64]. The drawbacks of smPCR also derived from single molecules are competing progresses and contamination. Therefore reducing the efficiency of side reaction during smPCR should be taken into consideration, including the design of primers, thermal cycling and selection of DNA polymerase [65]. Moreover, templates undergoing limiting dilution will conform to a Poisson distribution mode, that is, there exist a possibility that several wells contain more than one DNA molecule [66]. If DNA templates of these wells are variant in the beginning, the assessment of genuine mutations might be confused with thise resulting from bypass of chemically damaged nucleotides [65]. Based on smPCR, a modified single-molecule amplification named digital PCR appeared. It optimizes smPCR by changing the results examining step into automatable detection of fluorescence signals. Besides point mutations, digital PCR (dPCR) methods are also capable of detecting deletions. Belmonte et al. checked the ND4 gene which has great possibility of suffering deletion and the precise deletion proportion they discovered was 2.8% [67]. A linear regression model showed that the theoretical detection limit of deletion could be as great as approximately 1% by dPCR. Combining with microfluidics devices makes this technology a high-throughput method while analyzing low level heteroplasmy [68,69]. 

**Figure 5:**
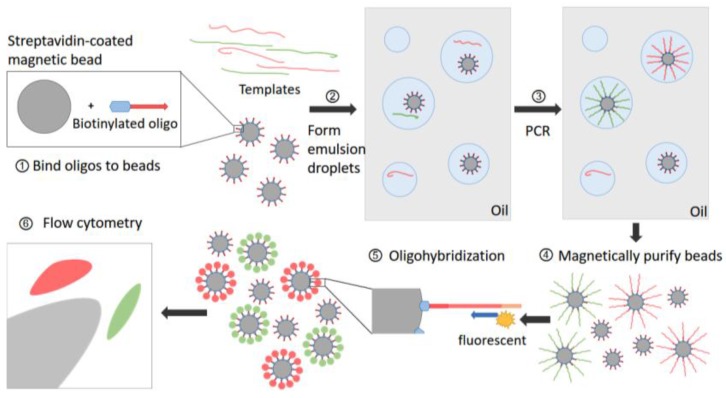
BEAMing procedure. Step 1: Magnetic beads coated with streptavidin are bound to biotinylated oligonucleotides (oligos). Step 2: An aqueous mix containing all the necessary components of PCR, beads and template DNA are stirred together with an oildetergent mix to create microemulsions. Red and green lines represent two kinds of templates which may differ from each other by one or more nucleotides. The aqueous compartments (blue circles in grey oil layer) are fully diluted to ensure each of them contain an average of less than one template molecule and less than one bead. Step 3: These microemulsions are subjected to thermal cycles of conventional PCR. If a DNA template and a bead are present to be together in a single aqueous compartment, the bead-bound oligos act as a primer for amplification. Step 4: Breaking the emulsions and the beads are purified with a magnet. Step 5: Fluorescently labeled primers are hybridized to the amplified target DNA molecules, which renders the beads as specific signal after appropriate laser excitation. Step 6: Flow cytometry is used to count the beads with different fluorescence signals.

Also with the aid of emulsion PCR, the size of dPCR reaction units can be narrowed down to picoliter-forming droplets, which means a great expansion of throughput. By fastening targeted oligonucleotides on the surface of magnetic beads, Vogelstein et al. devised a beads, emulsions, amplification and magnetics (BEAMing) system improving the detection limit tremendously (Figure 5). This system started from the magnetic beads coated with streptavidin then bonded to biotinyland oligos. An aqueous mix containing all the components for PCR including those beads was stirred together with an olidetergent mix to create proper sized microemulsions to make sure one or no bead was packaged in each droplet. Once these beads took on a specific fluorescence signal, they could be sorted out using flow cytometry [70]. At present, the BEAMing system is more applied to identify circulating DNA molecules due to its capability of detecting mutated DNA molecules even if outnumbered by wild-type DNA by a factor of 10,000 to 1 [71,72]. Although digital PCR optimized the procedure of original single-molecule PCR drastically, its disadvantages cannot be overlooked. Compared with smPCR, which can be seen as an improved amplification step of targeted reads before sequencing, dPCR can only detect recorded variations, so digital PCR may meet the needs of more applications of clinical or forensic situations, while smPCR is more suitable for discovering de novo mutations.

### 3.4. Next Generation Sequencing

In terms of identifying recorded heteroplasmic variations, plenty of methods can provide diverse choices according to the sample under study. PCR-based approaches mentioned before are available on small scale species otherwise they would result in heavy expenses and labor-abundant experiments. High-throughput sequencing, which is widely known as NGS, easily obtains the whole genome of species. Working as a synthesis-by-sequencing mode, NGS offers a higher throughput than older methods, providing the conditions for detecting low-level heteroplasmy in mitochondrial genomes. Although the proportion of heteroplasmy resulting in phenotypic appearance is as high as 70~90% [73], the possibility that high-level mutations arising from low level ones still exists, so the monitoring of some rare pathogenetic mutations seems to be essential. However, due to some features of next generation sequencing platforms, there are systematic errors which cannot be avoided. For example, the Illumina GA platform uses the same laser to excite A/C and G/T, producing similar emission spectra that leads to errors involving A/C and G/T [74]. These sequencing mistakes will disturb the identification of genuine mutations when it comes to low-level (5~20%) or rare heteroplasmy (0.5% or less) [75]. The most common measure to distinguish between a substantial signal and background noise is to perform control group experiments. In general, biospecimens without mitochondria like ρ0 cells [49] or bacteriophages [74] will be sequenced as control groups and the background error rate of amplification and sequencing platforms would be collected. Then a feasible heteroplasmy detecting threshold can be established according to the background noise rate [71]. Sometimes the estimation of thresholds is also assisted by computer emulations. With the aid of these in-silico virtual experiments, researchers are informed about the correlation between minor allelic frequency (MAF) and false-positive or false-negative percentages under different depths, making them useful for determining a proper threshold value [74]. Sometimes for authenticating the ability of distinguishing true mutations, artificial heteroplasmic mixtures are applied [74,76,77]. At any rate, more accurate quantitative analysis methods are still sought after. 

**Figure 6:**
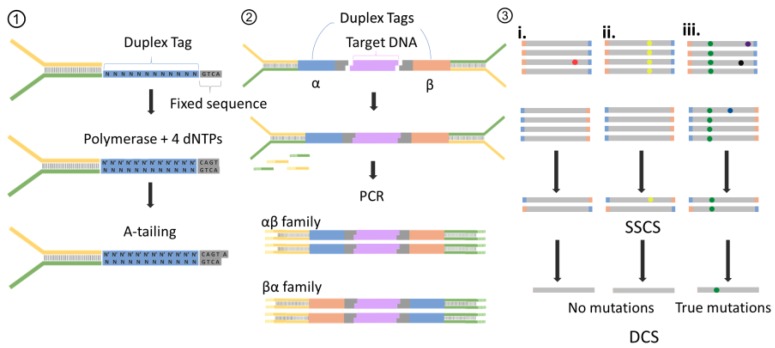
Procedure of duplex sequencing (DS). (1) Adapter synthesis. A 12 randomized base Duplex Tag is appended to one of two sequencing adapters, and then complete the complementary strand with DNA polymerase. Complete adapter A-tailing is ensured by extended incubation with polymerase and dATP. (2) Duplex sequencing working flow. Shared, T-tailed target double-stranded DNA molecule is ligated to A-tailed adapters. After PCR amplified with primers of adapter, two types of attachment are produced. Those derived from one target DNA fragment will have the α tag sequence adjacent to flow-cell adapter sequence 1 (yellow) and the β tag adjacent to flow-cell sequence 2 (green). (3) Error correction. Reads sharing a unique set of tags are grouped into paired families with members having strands labeled reciprocally (i–iii). (i) Mutation (red spot) present in only one or a few family members represent sequencing mistakes or PCR-introduced errors occurring late in amplification. (ii) Mutations (yellow spots) present in some or all the fragments of one family member but not in the other member. This situation represent that those mutation arise from PCR errors during the first round of amplification. (iii) When mutations occur on both strands of a DNA fragment appear in all members of a family pair, those are true mutations. Then reads with paired tags are grouped into single-stranded consensus sequences (SSCSs) with the bases of SSCSs fragments depend on the optimal base of each position. After that, SSCSs are reduced into duplex consensus sequences (DCSs).

Over the past five years, with the appearance of a barcode-based NGS technology named duplex sequencing (DS, Figure 6), the heteroplasmy detection threshold has been improved to 0.01% [78]. In addition to the adoption of the standard Illumina library preparation procedure, two distinct random sequences (i.e., tags of reads) were linked to the ends of targeted reads. After amplification of this attachment, each single strand molecule from the same double-stranded DNA fragment will be marked with the same pair of tags attached to reversed ends of those two single strand reads, which forms a pair of duplex sequences. While calling heteroplasmy, mutations that are detected 100 percent on both the duplex sequences are realistic. Ahn et al. utilized the DS method to develop an identification of nine novel mutations which were not reported before [75]. An analysis strategy of duplex sequencing results that removes the reliance of reference sequence was already deployed as a standalone application [79]. Single strand fragments were merged into families to form single-strand consensus sequence (SSCS) with a threshold that required a user-specific number of reads to produce a consensus. The consensus calling was conducted by determining the majority bases at every position which also meet a Phred quality score requirement of user-specific number. Next up, duplex consensus sequences were collected form SSCSs, and filtered with the removal of sequences harboring ambiguous nucleotides. Ultimately these DCSs were mapped against the reference genome and realigned to normalize gap-containing regions and the resulting alignments are applied to call variants [79]. Although sequencing mistakes occur in duplex tags and bring about misjudgment of the reads collection, duplex sequencing still demonstrates a breadth of application. This strategy provides threads of barcode-based quantitative methods going for some smaller-scale genomes like mitochondrial genomes. 

## 4. Discussion

Mitochondria are intergrown with eukaryotic cells, and play an important role in a considerable number of physiological processes. The behavior of mtDNA is quite different from the nuclear genome. Heteroplasmy of mtDNA hides pathogenetic mutations from regular detection which ought to be discerned as precisely as possible. Due to the mitochondrial threshold effect, phenotypic manifestation of the genetic defect occurs until the proportion of heteroplasmic variations reaches a threshold level, and threshold values of different tissues or pathologies are not identical [80]. For example, the threshold value of MERRF was 90% while MELAS’s was only 65% [80]. Thus, there exists a probability that the mitochondrial heteroplasmies are much more frequent than epidemiological estimates of the prevalence of mtDNA diseases. Confirmed by the whole-genome searching of mtDNA by Ye et al., 4342 heteroplasmies were found in 1085 individuals, with 89.68% of them harboring at least one heteroplasmy. The majority of these heteroplasmies were low-frequency (MAF < 5%) [81]. During cell division or fusion-fission cycles among mitochondria, the proportion of mutant mtDNA can vary and drift toward homoplasmy over time. Additionally, with increasing age or the accumulation of external damages, the percentage of heteroplasmic variations are likely to increase [82]. Even though dysfunctional mitochondria can be removed by mitophagy, the activity of that mechanism was observed to be reduced in aged endothelial cells versus young human ones [83]. For those mitochondrial diseases, some genetic therapies have focused on the change of nuclear genes which encode essential proteins for mitochondrial functions through the transfection of vectors, while other more promising strategies reduced the ratio of mutant to wild-type mtDNAs by inhibiting the replication or fusion of mitochondria [84]. In some heteroplasmy- affecting cases, human mitochondria harboring the MERRF mutation were able to internalize normal yeast tRNA to fix mitochondrial functions [85], and imported restriction endonucleases also helped transfect cognate normal genes into their specific sites [86]. Moreover, the pronuclear transplantation (PNT) method was used as a therapy of inherited mitochondrial diseases. After replacing the cytoplasm of normal cells, the percentage of mutant mitochondria was reduced to less than 2.0% [87]. PNT showed its potential for decreasing the risk of mitochondrial diseases, but it cannot prevent their occurrence. Therefore, detecting mtDNA heteroplasmies, especially those present at low-level frequency, can support the preventive diagnosis and treatment of diseases which haven’t shown any phenotypic manifestations yet. However, due to the tiny size of mitochondria and burdensome methods of extracting mitochondria from tissue samples, most of the strategies focus on isolating mtDNA directly or indirectly rather than only grabbing mtDNA. Several enrichment operations would be used prior to sequencing, including PCR or whole genome amplification. PCR-based methods might be the most popular ones. Among PCR amplification strategies, long-range (LR) PCR demonstrated relatively better uniformity derived from its lesser amount of amplicons and thus it is widely used. Nowadays, whole genome amplification is applied more and more frequently, especially for single cell analysis due to the fact it avoids using specific primers. Apart from amplification uses, PCR technologies are also applied for quantitative reactions. As for recorded mutations, the detection limit can be brought down by digital PCR to a factor of 10,000 to 1, and single-molecule PCR is able to discover unrecorded mutations although the positive results are appear as bright bands in the electrophoretograms which are very laborious to analyze. With the development of bioinformatics technology, collecting mtDNA data from exon byproducts or RNA sequencing was shown to be feasible, but researchers have to consider the impact of homologous sequences in nuclear genomes (nuMTs). As for heteroplasmy calling, nowadays high-throughput sequencing provides higher use rates with Sanger sequencing and quantitative PCR strategies acting as the gold standards for heteroplasmy decisions. Because of the high-throughput feature of NGS, mistakes caused by amplifications or base-calling steps hinder the analysis of true mutations, especially for fractional amount of samples like cell free DNA molecules. Several strategies could be implemented to enhance the specificity of targeted products as well as improve the accuracy of detecting true HFs, for instance, grouping reaction conditions with higher specificity or modifying alignment schemes of mitochondrial genomes. Moreover, studies on mtDNA mutations are too numerous to count, while the linkage studies of those variations are few. In the investigation of tissue-related mtDNA heteroplasmy, Li et al. discussed the co-occurrence of nine pairs of heteroplasmic sites [18], but the study did not go further due to the small sample size, and lacked sufficient power to detect more associations. The further investigations of interactions between different heteroplasmies even of the whole mitochondrial genome heteroplasmic linkages are valuable.

## 5. Conclusions

Totally speaking, the detection of mtDNA heteroplasmy and HF determination are dominated by NGS technology at the present, supplemented by other quantitative means to verify the credibility of its results. In the future, the studies of mtDNA heteroplasmy may pay more attention to the single-cell level and focus on the linkage of mutations.

## Figures and Tables

**Table 1:** Comparison of heteroplasmy detection methods.

Methods	Detection Limit	Variations Type ^2^	
		SNPs	Indels
Sanger sequencing	~15%	Y	Y
Polymorphism ratio sequencing	~5%	Y	Y
ARMS-qPCR	~0.1%	Y (recorded)	N
Single-molecule PCR	~7% [53]	Y	Y
Digital PCR	~0.01% ^1^	Y (recorded)	Y (recorded)
High-Throughput Sequencing	~0.1%	Y	Y

^1^ ~1% for deletions. ^2^ we marked Y for yes and N for no. The word ‘recorded’ represents that this method can only detect the present variations which are recorded in datasets. The absence of ‘recorded’ means both of the recorded and novel variations can be detected.
